# A three-dimensional high throughput assay identifies novel antibacterial molecules with activity against intracellular *Shigella*

**DOI:** 10.1038/s44259-025-00110-6

**Published:** 2025-05-15

**Authors:** Voong Vinh Phat, Andrew Shih Teong Lim, Cristina De Cozar-Gallardo, Maria Isabel Castellote Alvaro, Demetrio Muñoz Alvarez, Elena Fernandez Alvaro, Lluis Ballell-Pages, Sonia Lozano-Arias, Stephen Baker

**Affiliations:** 1 The Hospital for Tropical Diseases, Wellcome Trust Major Overseas Programme, Oxford University Clinical Research Unit, Ho Chi Minh City, Vietnam; 2 The ALBORADA Drug Discovery Institute, University of Cambridge, Cambridge, United Kingdom; 3 GSK Global Health, Tres Cantos, Madrid, Spain; 4 Molecular Modalities Discovery, GSK, Tres Cantos, Madrid, Spain; 5 A*STAR Infectious Diseases Labs (A*STAR IDL), Agency for Science, Technology and Research (A*STAR), Singapore, 138648 Singapore

**Keywords:** Drug discovery, Gastrointestinal diseases, Infectious diseases, Microbiology, Antimicrobials

## Abstract

The Gram-negative bacterial species *Shigella* is the second leading cause of diarrhea among children in low and middle-income countries (LMICs) and is a World Health Organization (WHO) priority pathogen. *Shigella* infections are becoming increasing difficult to treat due to antimicrobial resistance (AMR), leading to an urgent need for new antimicrobial agents with novel modes of action. *Shigella* pathogenesis is largely intracellular and antibacterial chemicals that preferentially work inside cells may be desirable to limit collateral AMR and block key components of the *Shigella* infection cycle. Aiming to facilitate the process of identifying antibacterial chemicals that kill intracellular *Shigella*, we developed a high-throughput screening (HTS) cell-based chemical screening assay. The three-dimensional (3-D) assay, incorporating *Shigella* invasion into Caco-2 cells on Cytodex 3 beads, was scaled into a 384-well platform for screening chemical compound libraries. Using this assay, we evaluated >500,000 compounds, identifying 12 chemical hits that inhibit *Shigella* replication inside cells. This simple, efficient and HTS-compatible assays circumvents many of the limitations of traditional screening methods with cell monolayers and may be deployed for antibacterial compound screening for other intracellular pathogens.

## Introduction

Shigellosis is an acute enteric infection caused by organisms belonging to the genus *Shigella*. With 165 million cases per year globally and an estimated 1.1 million deaths, *Shigella* remains a significant public health challenge^1^. Data from the Global Enteric Multicenter Study (GEMS) found that *Shigella* was a major contributor to the global diarrhea burden and the most common aetiological agent in young children with diarrhea^2^. Currently, there is no licensed vaccine to provide protection against any *Shigella* species^3,4^. Consequently, antimicrobials are the mainstay for disease control and are commonly used to treat severe *Shigella* infections^1^. However, *Shigella* are highly adept at acquiring multidrug resistance (MDR) plasmids from other Enterobacteriaceae, and antimicrobial resistance (AMR) mutations are commonly associated with successful lineages^5,6^. Treatment options for *Shigella* infection are rapidly diminishing due to resistance to the majority of commonly used antimicrobials, included those recommended by the WHO as empirical choices for *Shigella-*related diarrhea^7^.

A universal increase in AMR necessitates the need for the discovery of new antibacterials, ideally with novel modes of action. However, antibacterial drug discovery and development is challenging^8^, as exemplified by various notable examples in the pharmaceutical industry^9,10^. Such issues have also been compounded by the “innovation” gap where no new classes of antibacterial agents were discovered^11,12^. One of the key discovery issues is finding small molecules that can overcome the unique architecture of the bacterial cell wall. The bacterial outer membrane of Gram-negative bacteria contains outer membrane proteins and porins which create a hydrophilic barrier. The hydrophilic barrier contrasts the lipophilic barrier that characterizes the mammalian cell membrane, which antibacterial compounds must overcome to target intracellular bacteria^13^. Therefore, antibacterial compounds should ideally have physicochemical properties that allow permeation into mammalian and bacterial cells. Additionally, there is discussion regarding the physicochemical space that antibacterial agents occupy, which may differ to those occupied by alternative drug classes^14,15^. Phenotypic cellular approaches for screening new antibacterial agents have the advantage of being able to identify compounds with intracellular activity early in the process, although this approach has the risk of a lower hit rate due to the more restrictive physicochemical property requirements.

Caco-2 cells are widely used in intestinal epithelial cell-based models to evaluate and predict the absorption of compounds due to their simplicity and reproducibility^16^. However, experiments with Caco-2 cells are normally conducted on permeable filter inserts or collagen-coated surfaces and experimented^17,18^, which is low-throughput and not amenable to screening large numbers of compounds across a broad chemical space. Given the limitations of traditional methods, micro-carrier technologies are being exploited to grow various anchorage-dependent cell types that are not able to grow in a suspension or cells that are adherent but need to be differentiated for functionality^19^. Micro-carrier technology has multiple advantages over more traditional approaches, including enhancing the surface-volume ratio, reducing experimental time, increasing reliability and permitting the use of adherent cells like a suspension system and scale-up can be performed in bioreactors^20–22^. Notably, Caco-2 cells cultured on microcarrier beads have been applied for screening chemicals against a range of pathogens, but have not yet been deployed for *Shigella*^19,23–25^.

With the aim of identifying new compounds with intracellular killing activity against *Shigella*, we developed a three-dimensional Caco-2 cell platform to perform in vitro phenotypic high-throughput screening of >500,000 compounds against *Shigella*. Caco-2 cells were cultured in high-yield on Cytodex 3 beads and were then infected with nanoluciferase-producing *Shigella flexneri*. The resulting infected Caco-2 cells were then used for screening. We aimed to evaluate the efficiency of this method and apply this new assay to early-stage drug discovery. Our results confirmed that this new assay is a comparatively simple method in which to identify compounds with antibacterial properties against intracellular *Shigella* without the limitations of existing monolayer models.

## Results

### High-throughput screening (HTS) assay development

**Fig. 1:**
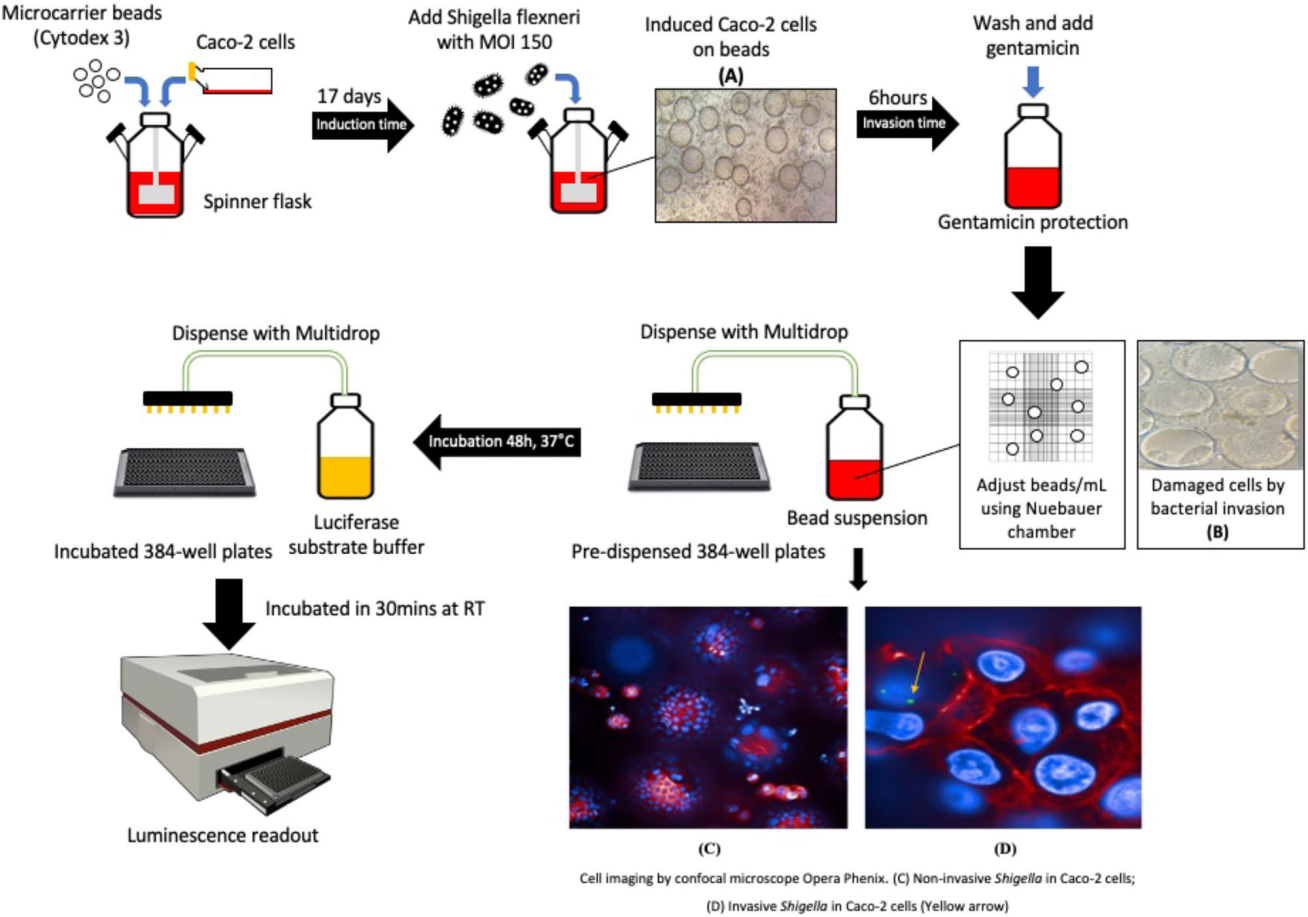
High-throughput screening of Caco-2 cells. Light microscope images of Caco-2 cell grown on Cytodex 3 beads (**A**) Induced cells on beads, (**B**) Cell damaged and detached off beads. Cell imaging by confocal microscope Opera Phenix (**C**) Non-invasive in Caco-2 cells, (**D**) Invasive Shigella inside Caco-2 cells. Components of this figure was generated by the authors using AI tools (ChatGPT/DALL·E, OpenAI) and is original content. The author retains all rights.

We aimed to establish a high-throughput screening assay based on the Caco-2 cell model to identify potential drug targets that could inhibit the replication of *S. flexneri* inside cells (Fig. 1). *S. flexneri* infectivity was conducted using a 3-dimensional model of large intestine Caco-2 cells, which were grown in a bioreactor at the given cell and microcarrier concentrations in a humid atmosphere of 5% CO_2_ at 37 °C, 3-D cells were then harvested and transferred to cell-culture plates for experimenting or screening. The assay was optimized using a large-volume spinner flask (Wheaton, US), as an alternative to the rotating wall vessel (RWV) bioreactor as has been used in previous studies^20–22^. This vessel was used because it facilitated medium change during differentiation process, and scale-up of culture volume to make the assay amenable to HTS^26^, while not affecting the integrity of the Caco-2 cells, as evidenced by the analysis of sucrase, alkaline phosphatase (ALP) production, and ZO-1 formation, which are markers of Caco-2 functionality. Sucrase, which is an important digestive enzyme secreted in the small intestine, increases during differentiation of the enterocytes and is considered a reliable indicator of Caco-2 cell differentiation in vitro. During differentiation of Caco-2 cells, total activity of sucrase in the monolayer increased by 2.8-fold above baseline, and the activity relative to the 3-D model increased by 3.5-fold (Supplementary Fig. 1). Intestinal alkaline phosphatase is a digestive brush-border enzyme, which is highly upregulated during small intestinal epithelial cell differentiated. ALP activity demonstrated an increasing trend from day 8 to day 21 in both the monolayer and 3-D model (5.5-fold) (Supplementary Fig. 1). These increases were consistent with the induction of sucrase and ALP that has been reported for Caco-2 cells^27,28^. Both sucrase and ALP assays showed no significant difference (*p* value > 0.05, Mann-Whitney test) between both platforms over 21 days postconfluence (Supplementary Fig. 1). Additionally, ZO-1 proteins were expressed abundantly in the Caco-2 cells in both the monolayer and 3-D models, forming a clear, narrow, punctate line along the apical surface of cells (Supplementary Fig. 2). The presence of ZO-1 indicated the formation of tight junctions in cell-to-cell interaction^27,29,30^.

The optimization of assay parameters, such as invasion incubation time, the optimal time and multiplicity of infection (MOI) for *Shigella* invasion were determined using the balance between the Z’ factor, a statistical characteristic for comparison and evaluation of the quality of HTS assays^31^, and percentage of bacterial coverage (Supplementary Table 1). A MOI of 150 and six hours of invasion were determined to be the optimal assay conditions, which ensured 100% bacterial growth in wells, while maintaining the robustness of the assay (Z’ > 0.4; and S/B value > 2)^32,33^. Cell detachment resulting in empty beads were observed after four hours of invasion, this phenomenon was observed to increase with time. At 4 and 6 h of invasion, ~80–90% of beads had attached Caco-2 cells in comparison to pre-invaded beads. Induction was not suitable for screening after 6 h of invasion. Other testing conditions did not meet validation standards. MOIs of 5 and 10 were suboptimal as they required too much time for invasion and the robustness of the assay could not be maintained. An MOI of 100 demonstrated improved performance with perfect coverage after six and eight hours of invasion; however, the Z’ factors were not stable and fell out of standard range (Z’ < 0.4). To reduce the number of beads required for primary screening, three different bead concentrations (1000, 2000 and 4000 beads/ml) were tested, but only the 4000 beads/ml gave a suitable for a high-throughput screening assay (Z-score (mean Z’ = 0.57) and S/B values > 2-fold).

We evaluated the invasive efficiency of *S. flexneri* by defining invasive efficiency as the percentage of intracellular bacteria (CFU/ml) to total number of bacteria (CFU/ml) (%) at the same duration of invasion (Supplementary Table 2). The *S. flexneri* SF_nanoluc strain, *S. flexneri* serotype 2a 2457 T carrying reporter plasmid pMK-RQ_tac+nanoluc (Supplementary Table 3), with an invasion efficiency of 0.083%, exhibited the highest invasion efficiency at six hours of invasion. This score resulted in approximately eight invading bacteria for every 10^4^ CFU input. In terms of bacteria per cell or bead, we estimated seven intracellular *S. flexneri* for every 100 cells or ~15 *S. flexneri* for each bead.

We next assessed dose-response potential of the assay by measuring the intra-assay and inter-assay of 11 different commercially available antimicrobials (Supplementary Table 4). Intra-assay coefficient of variation (CV) is a measure of the variance between data points within an assay, represented by sample replicates within the same plate. Inter-assay CV is a measure of the variance between runs of sample replicates on different plates that can be used to assess plate-to-plate or batch-to-batch consistency. IC_50_ values were used to calculate coefficient of variances in percentage (Supplementary Table 5), then we determined the repeatability and reproducibility of this Caco-2 cell-based assay in case of maintained robustness of the assay. The intra-assay CV and the inter-assay CV across 11 drugs ranged from 0.16% to 8.53% and from 1.43% to 14.82%, respectively. The intra-assay CV ( < 10%) demonstrated a high degree of reproducible in each batch, and the inter-assay CV ( < 15%) demonstrated acceptable reproducibility between batches of the cellular assay.

The IC50 comparison between plate-based (monolayers) and bead-based assay was applied for control (Moxifloxacin) to validate and compare between two methods. The average IC50s from monolayer assays were lower than bead-based assays, but there was no significant difference (*p* value > 0.05, using Mann-Whitney test) between two methods (Supplementary Table 6).

### High-throughput screening process validation

A validation set of small molecules, comprised of ~10,000 molecules, representing a wide diversity of chemotypes present within the GSK chemical collection, was used to evaluate the HTS assay configuration. The assay displayed an average Z’ factor of 0.48 and the obtained cut-off (average response of the sample distribution +3 standard deviations (SD)) was 48.87%. The chemical hit rate was ~2.5% (247 active compounds), but signal patterns were detected after analysing the dataset by using ActivityBase software, signifying the errors and noise of dispense patterns caused by unevenly dispensed beads. The discordant plates were re-run.

With the aim to identify lead compounds with novel modes of action, we performed structural analysis on active compounds, discarding 43 compounds that belonged to known antibacterial classes, including dihydrofolate reductases (DHFR), quinolones, and bacterial topoisomerase (BTIs). We then performed a dose-response assay on the 204 remaining compounds. From the four compounds that gave dose-dependent response against intracellular *S. flexneri*, three of them were active in both replicates and had IC_50_ < 10 μM and the remaining chemical was active in one of two replicates with an IC_50_ = 36.31 μM. These compounds were further tested for cytotoxicity in HepG2 cells and were subjected to physicochemical characterization (Supplementary Table 7).

### HTS primary screening campaign

**Fig. 2:**
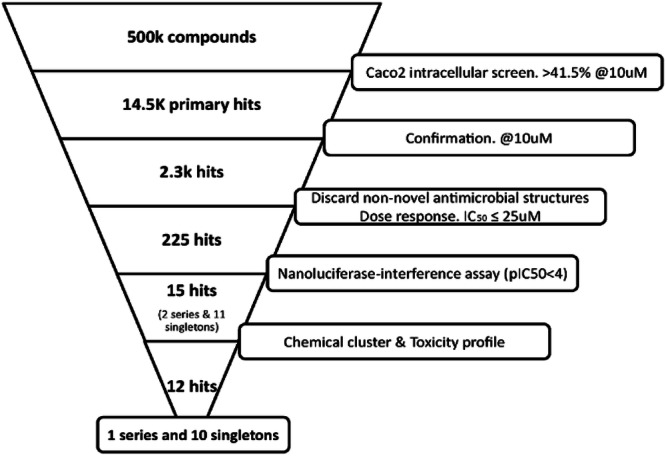
Study flowchart. Diagram outlines the experimental screening process leading of 500,000 compounds to confirming 12 potential antibacterial hits against intracellular *Shigella*.

The primary screening campaign of 518,282 compounds was performed in different 49 runs utilising 1521 individual plates, the workflow is presented in Fig. 2. The HTS assay was conducted using the optimised method, which was the 3-D Caco-2 cells infected with *S. flexneri* SF_nano strain at an MOI of 150 in six hours, followed by gentamicin protection and subsequently diluted to the final assay concentration of 4000 beads/ml. For the HTS primary screen, 25μl of the infected 3-D Caco-2 cells at 4000 beads/ml was dispensed into prepared plates for screening. Overall, 45 discordant plates were re-run because their Z’ factors were <0.4 (Supplementary Fig. 3). The robustness of the assay was maintained with an average Z’ factor of 0.55. Compounds with inhibition rates greater than the robust cutoff value of 41.48% were considered active, with 14,451 compounds selected. With the consideration of false signal patterns (systematic errors)^34^, 14,451 primary hits were screened again for confirmation at 10 μM in the same assay format, representing a hit rate of 0.44% (2,283 compounds). After structural analysis of hit compounds, we discarded 221 non-novel antimicrobial compounds, including BTIs, macrolides, quinolones, and DHFRs. The final list of 2062 compounds were progressed into a dose-response assay, removing those with IC_50_ > 25 μM. As a result, 225 compounds exhibited inhibition with IC_50_ ≤ 25 μM, but 15 compounds were not accessible. Therefore, to factor out compounds that may be false positives due to nanoluciferase inhibition, 210 available preliminary hits advanced from the intracellular survival screen were tested in a nanoluciferase-interference assay in dose-response assays starting at 100 μM. Ultimately, we identified 15 compounds with a pIC50 < 4 and a pIC50 ratio between the Caco-2 cell assay and nanoluciferase interference assay >1, which indicated that no extracellular-nanoluciferase activity were identified to have intracellular activity against *S. flexneri* in this assay format (Fig. 2).

**Fig. 3:**
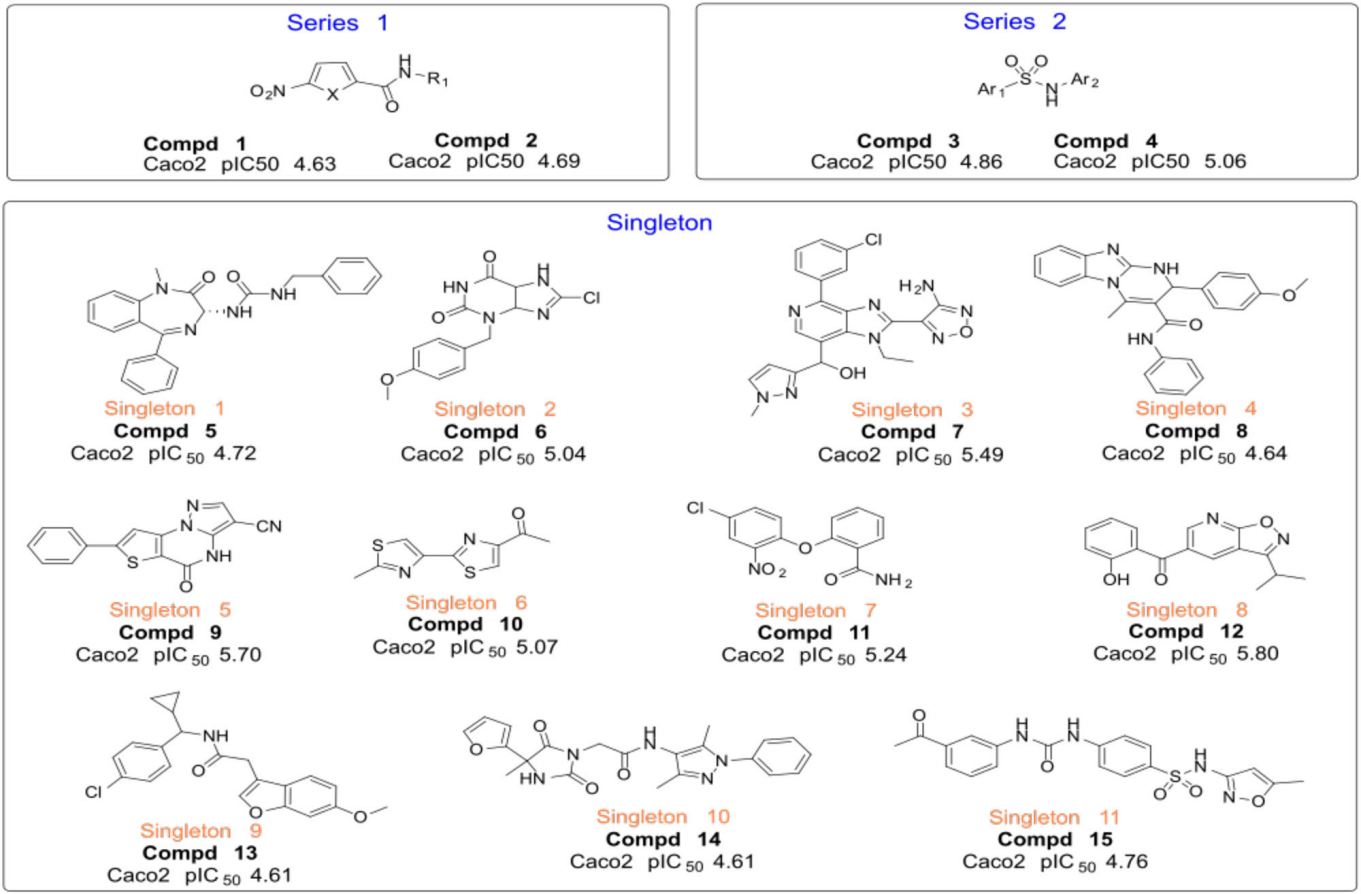
The identified antibacterial hits from the HTS campaign. Images show the chemical structure of the 11 singleton and two chemical series that were selected for additional profiling.

Upon chemical review, the 15 hit compounds were reorganized based on structure similarity into 13 clusters. Two chemicals were considered as a series, and the remaining 11 clusters were singletons (Fig. 3). To ensure that the compounds were suitable starting points as quality hits and to determine the toxic potential of the new chemical entities, an inspection of the compounds for structural features that may impact toxicity was conducted, followed by in vitro cytotoxicity testing on HepG2 cells. Four of the 15 structures carried an NO_2_ moiety, including two hits of Series 1, one hit of Series 2, and Singleton 7. Although the antibacterial activity of nitro-containing molecules is known to be broad, nitro groups have been extensively associated with mutagenicity and genotoxicity. Therefore, the compounds that were representative of the entire clusters were additionally tested on HepG2 cells, four compounds (1, 2, 8, and 11) were found to exhibit HepG2 cytotoxicity. Series 1 (compounds 1 and 2) and singleton 4 (compound 8) demonstrated some concerns of progression considering its selectivity (Caco-2 pIC50 vs HepG2) and its structural alert (Supplementary Table 8). The remaining 12 compounds (Table 1), represented by Series 2 and Singletons 1–3, 5, 6, and 8–11, were of significant interest as starting points for further optimization toward the drug discovery for the treatment of shigellosis.

**Table 1:**
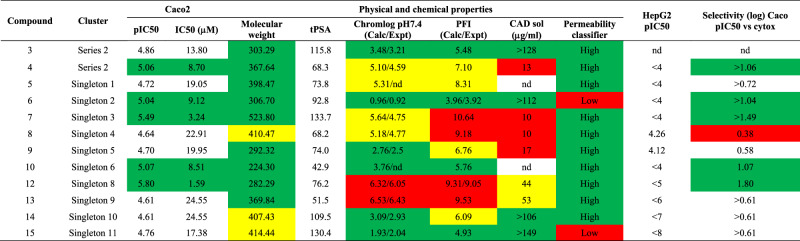
Complete profile of the selected compounds

Key: Green: MW ≤ 400; Chromlog ≤4; PFI ≤ 6; Permeability high, pIC50 ≥ 5; Selectivity ≥1; CAD solubility ≥100. Yellow: MW 400–500; Chromlog 4–6; PFI 6–9; Permeability medium; CAD solubility ≥ 30–100. Red: MW ≥ 450; Chromlog ≥6; PFI ≥ 9; Permeability low; Cytotox HepG2 ≥ 4.5; Selectivity ≤0.5; CAD solubility ≤30.

## Discussion

Pathogenic bacteria have been engaged in a sustained ‘arms race’ with antimicrobials since their introduction and antimicrobial-resistant *Shigella* has emerged as a significant global threat to public health^7^. Novel treatments and approaches are urgently required to combat infections caused by MDR *Shigella*. To aid in addressing this issue we developed, validated and executed an HTS Caco-2 cell-based system to screen a large compound library against *S. flexneri*. Compared to traditional cell monolayer screening, this assay system retained the disease-relevant cellular architecture, which improves the success of translating hits from the screen to cell-based or in vivo infection models. Additionally, this approach simultaneously has the common advantages of all HTS systems, which include scalability, amenability to automation, simplicity and rapid transfer of reagents.

Our project was initiated by establishing an HTS assay using a spinner flask, and experiments were performed in a 384-well format. We demonstrated that there was no significant difference in the structural integrity of the Caco-2 cells between microcarrier beads (Cytodex 3) and the monolayer format; *S. flexneri* still demonstrated invasion and spread from cell-to-cell in both systems. One of the main advantages of a bead-based system is a large surface area to grow cells to high density. This approach facilitates scale-up and reduces the floor space and incubator volume required for a given-sized manufacturing operation and eases the precise process control in a large-scale bioreactor. Consequently, the number of compounds screened per batch can be easily scaled up. However, we found that even after optimising conditions, bacterial invasion rates were substantially lower in comparison to the monolayer format^35,36^. Notably, not every Caco-2 cell was invaded by a *Shigella* organism and not every microcarrier bead possessed an invaded cell. Therefore, we compensated for this lower rate of invasion by increasing the number of beads to ensure each well contained intracellular *Shigella*.

In generating this new high-throughput approach we observed a high false discovery rate or chemicals that did not show a dose-dependent-response. After the primary screen, 2.79% of chemical were retained, with only 0.44% remaining after rescreening. This discrepancy may have been caused by false signal patterns recognized throughout the screen, such as edge effects (caused by evaporation), patterns on a plate in general, and errors in adding assay components (dispenser distribution). In addition, there were compounds, which were later found to inhibit the luciferase reporting system. Nonetheless, we employed various orthogonal screenings to filter out false positives. We next analysed what chemical scaffolds had been elucidated by our screening effort. To ensure that the compounds were suitable starting points for qualifying hits, we characterized the potential toxicity of the new chemical entities. As a result, 12 compounds represented by Series 2 and Singletons 1–3, 5, 6, and 8–11 were prioritized. Considering their overall profile in terms of physicochemical properties (most of them with predicted high permeability and different range of solubility) and initial toxicity profile, although the potency of these compounds was low (pIC50 values between 4.6 and 5.8), they could be considered as novel starting points for drug development against *Shigella* (Table 1). Additional research is currently being performed to assess these hits and convert them into developable hit series. Furthermore, these compounds also have the potential to be developed into tool compounds for antibacterial agents with preferential activity against intracellular bacteria. The validation of these types of tool compound will aid in the future design of novel antibacterial agents with intracellular activity. Additionally, various studies can be planned using such tool compounds to better understand the mode of action of these new chemicals.

In summary, we have shown that it is possible to integrate three-dimensional cell culture into an assay system that is amenable to HTS using microcarriers. We have demonstrated the capability of this system in an HTS of >500,000 compounds and have identified novel hits that have activity against intracellular *S. flexneri*. The experience in developing this assay system has enabled us to gain insight on assay optimization issues of which we have demonstrated steps to mitigate them. We think that the microcarrier-based assay system has utility in HTS to drive hit discovery programmes, and we envisage this assay system to be used in other intracellular bacterial infection models to pioneer hit discovery against gastrointestinal pathogens.

## Materials and methods

### Bacterial strains, cell lines, growth media and reagents

The bacterial strains, cell lines and plasmids used in this study are listed in Supplementary Table 3. *S. flexneri* isolates were routinely cultured on Luria-Bertani (LB) broth or LB agar. Where required, the growth medium was supplemented with antimicrobials at the following concentration: Kanamycin (50ug ml^-1^) for *S. flexneri* serotype 2a 2457 T (ATCC® 700930™) carrying plasmid pMK-RQ_tac+nanoluc (SF_nanoluc), and ampicillin (100ug ml^-1^) for *S. flexneri* ATCC 12022GFP.

Caco-2 cells were grown in 5-layer flasks (Nunc/Thermo Fisher Scientific) with basic EMEM (Sigma-Aldrich) supplemented with 10% FBS (Gibco), 1% L-glutamine in a humidified atmosphere of 5% CO_2_ at 37^o^C. The medium was changed every 72 h until 80–90% confluent layers were generated.

### Generation of the reporter strain

The synthetic gene Tac+Nanoluc (593 bp) was assembled from synthetic oligonucleotides and the fragment was inserted into the pMK-RQ vector, yielding the 18ADIM2C_Tac+Nanoluc_pMK-RQ (KanR) construct (hereafter referred to as pMK-RQ-nl). *Escherichia coli* K12 DH10B™ T1R was used for the propagation of the plasmid. Plasmid DNA was purified from transformed colonies and sequenced (Dynamimed, Spain) for verification. This process was conducted by contract to Invitrogen (Thermo Fisher Scientific, USA). Electroporation of pMK-RQ-nl into *S. flexneri* 2457 T was performed according to a standard protocol (NEB Electroporation protocol, C2986) yielding the *S. flexneri* serotype 2a 2457T-nl (or SF_nanoluc) used in this study.

### High-throughput screening assay

*S. flexneri* SF_nanoluc was grown overnight on a 50mgL^-1^ kanamycin LB plate at 37 °C. Bacteria were scraped from the plate surface and sub-cultured in warm supplemented EMEM medium for 2 h (at 37 °C and 200 rpm) and diluted to get OD around 0.3 at 625 nm. Induction was performed in 500 ml or 1000 ml siliconized spinner flasks under closed culture conditions. 200 mg autoclaved Cytodex 3 beads (GE Healthcare), rehydrated with cation-free PBS (Sigma), were washed with supplemented EMEM medium and inoculated with 1.7 million Caco-2 cells/ml in half of the desired culture volume of the medium. The spinner vessel was kept static for 4 h in a humidified atmosphere of 5% CO_2_ at 37 °C, allowing Caco-2 cells to bind to beads. The remaining medium was added into the vessel and stirred at 32.5 rpm. The medium was changed every 72 h, and the induction was used on day 17 to ensure Caco-2 cells were fully differentiated.

Cell concentration induction was measured by NucleoCounter® NC-100™ (ChemoMetec Version 2.4). Cellular invasion was initiated by adding the bacterial stock into cell induction with optimal MOI, and the vessel was stationary for an hour before stirring at 22.5 rpm for 5 h in a humid atmosphere of 5% CO_2_ at 37 °C. After incubation, culture media was discarded by centrifugation; and a gentamicin protection assay was performed, in which cell beads were washed twice with a warm medium and EMEM containing gentamicin (100ug ml^-1^) was added to the chamber of the system. The cell culture was incubated for 1 h with stirring and then washed three times with a warm medium before 25 μl of the invasive mixture was dispensed into each well. Plates were incubated for two days in a humid atmosphere of 5% CO_2_ at 37 °C before proceeding to develop the luminescent signal by adding 10 μl mixture of nanoluciferase substrate (Nano-Glo® Luciferase Assay System, N1130, Promega) into each well. After standing at room temperature for 30 min, luminescent signals were quantified by the Envision Reader (PerkinElmer) in focus luminescent mode. For the bacterial tracking, we used the green fluorescent protein in *S. flexneri* ATCC 12022GFP to monitor the invasion. A confocal HCS microscope (Opera by PerkinElmer) was used to confirm the invasion of *S. flexneri* into Caco-2 beads (Fig. 1).

### Compounds and assay plate preparation

The HTS assay was performed in 384-well black clear-bottom plates (Greiner). Screening compounds were prepared with an appropriate DMSO concentration (0.5% in cell-based assay). Every assay plate contained 16 wells of DMSO as negative controls and 16 wells of moxifloxacin at a final concentration of 20 μM as positive controls. These controls were used to monitor assay quality through determination of Z’ factor as well as normalizing the data on a per-plate basis.

To assess the quality of the dose-response assay, 11 commercially available antimicrobials were used to evaluate the reliability of the HTS assay. The percentage of coefficient of variation (CV%) was calculated by accessing the IC50 (μM) deviation of selected positive controls. Intra-assay reproducibility was determined by comparing the IC50 that were generated in the same run of the two replicates. Inter-assay reproducibility was assessed by comparing the IC50 generated by two replicates of each day over two days. Intra-assay %CV should be <10%, while inter-assay %CV should be <15%. To validate the HTS assay configuration, a set of 10 thousand compounds was assayed in 384-well plates at a final concentration of 10 μM. Assay were run in duplicate and confirmed if needed, a validation data was assessed to analyse the pros and cons of the whole procedure.

The primary screening campaign of 520,000 compounds was screened at the single shot of 10 μM final concentration; active compounds were retested at the same concentration (10 μM) again to confirm their activities. Following screening, all non-novel structures were removed, and the remaining compounds were subjected to a dose response (DR) assay, which was set as 3-fold serial dilution started at 100uM each compound. Those compounds that had DR to targeted organism of IC50 ≤ 25 μM were progressed to toxicity and structure analysis.

To select the most drug-like compounds, Fasted State Simulated Intestinal Fluid (FaSSIF) is used to test the dissolution and permeability of compounds, as well as their stability in the gastrointestinal tract^37^. Human ether-à-go-go-related gene (hERG) is the gene that encodes a potassium channel protein, which is essential for normal heart rhythm. It is important to screen compounds for hERG blockade^38^.

### Optimizing invasion conditions

To determine the optimal condition (multiplicity of infection - MOI and invasion time) for the invasion process, the induction products were infected with SF_nanoluc at an MOI ranging from 5–150 (5, 10, 100, 150) for five time points (4 h, 6 h, 8 h, 10 h and >16 h). After gentamicin protection, 25μl of invasion was dispensed into two prepared 384-well plates (32 wells negative control with DMSO and 16 wells positive control with 20μM moxifloxacin) the number of beads >5000 beads/ml. The experiments were repeated three times. Results were assessed by Z’ factors (Z’ ≥ 0.4)^33^, percentage of bacterial growth in wells, and bead coverage observed by microscopy. If the coverage of beads with cells was <70%, the experiment was not carried out any further (data not show). For the percentage of bacterial growth, the results were determined by plating on 50mgL^-1^ kanamycin LB plates. If SF_nanoluc strains fully grew in all negative control, it was defined as “perfect coverage” at its tested condition.

To increase the number of plates per run, the number of beads per well was optimized with three different bead concentrations (1000, 2000, and 4000 beads/ml). 10μl homogenized invasive mixture was dropped on each counting grid of a Neubauer chamber, and the number of beads (with or without attached cells) were enumerated by microscopy. We repeated three times and calculated the average before dispensing into each well of prepared plates. The effects of bead concentration on Z’ factor were considered in deciding the concentration of beads to be used in the high-throughput screening assay.

### Invasion efficiency evaluation

A bacterial quantification assay was adapted and modified from a previous study to calculate the invasion rate of *Shigella* into Caco-2 cells^39^. After inoculation, 50 ml homogeneous invasion was withdrawn at three time points (2 h, 4 h, and 6 h) and equally divided into two tubes (A and B) at each time point. Tube A went directly to cell lysis. After being washed with fresh medium and centrifuged at 8000 rpm for 10 min twice to collect all beads and bacterial pellets, the mixture was resuspended in 5 ml of PBS and divided into five different 1.5 ml tubes (1 ml/tube). One was used for cell counting and another for bead counts. The remaining three tubes were lysed with 0.1% saponin in one hour and the dilutions of lysate were plated on 50mgL^-1^ kanamycin LB plates for quantification. Tube B was used to determine bacterial invasion; extracellular bacteria were killed with gentamicin (100mgL^-1^) for 1 h. Then cultured beads were washed twice with PBS at 800 rpm and re-suspended in 5 ml PBS. Then, the same process of tube A was applied. The experiments were repeated four times. Invasion rates were calculated as the percentage of alive *Shigella* after gentamicin protection compared to the total number of *Shigella* before being treated with gentamicin.

### Sucrase assay

Sucrase is an important digestive enzyme secreted in the small intestine on the brush border and catalyses the hydrolysis of sucrose to its subunits of fructose and glucose. Sucrase is increased during differentiation of the enterocytes and is considered a reliable indicator of Caco-2 cell differentiation in vitro^40,41^. We monitored sucrose production at day 8, 12, 15 19 and 21 after seeding, in the Cytodex 3 beads system as well as in monolayers. Measurements were conducted by using the fluorescent Amplex® Red Glucose/Glucose Oxidase Assay Kit (A22189, Invitrogen, Molecular probes) following the manufacturer’s instructions.

### Alkaline phosphatase (ALP) activity

The intestinal alkaline phosphatase encodes a digestive brush-border enzyme, which is highly upregulated during small intestinal epithelial cell differentiation^42^. ALP activity was measured in the supernatant of Cytodex 3 and monolayer Caco-2 cells during differentiation 8, 12, 15, 19 and 21 days after seeding. Measurements were performed by using the Alkaline Phosphatase Assay Kit Colorimetric (Abcam, ab83369) kit according to the manufacturer’s instructions.

### Assessment of the tight junctions of Caco-2 cells

The primary functions of tight junctions (TJ) in epithelial cells are to create a regulated barrier in the extracellular space, to regulate the passage of ions and molecules between cells, and to mark the division between apical and basolateral surfaces of cells in cellular differentiation^43^. TJs are essential for the polarization of epithelial cells. Intracellular transduction pathways can regulate the integrity of the tight junctions. We identified and characterized ZO-1 as a peripheral membrane protein specifically associated with the cytoplasmic surface of tight junctions. We observed immunofluorescence of human Caco-2 cells stained with mouse anti-ZO-1 Monoclonal Antibody - Alexa Fluor® 488 (Product #339188, Thermo Fisher Scientific). DNA is counter-stained with blue Hoechst 33258 (Product #H3569, Thermo Fisher Scientific). Immunofluorescence analysis of ZO-1 was performed using 90% /100% confluent log-phase Caco-2 cells in the beads system. Cells were fixed with 4% paraformaldehyde for 30 min, permeabilized with 0.1% Triton™ X-100 for 10 min and blocked with 1% BSA for 30 min at room temperature. The cells were labelled with ZO-1 Monoclonal Antibody (ZO1-1A12), Alexa Fluor 488 at 5 µg/mL in 0.1% BSA and incubated overnight at room temperature. Nuclei were stained with DAPI at 1ug/mL concentration without washing.

### Interference with nanoluciferase reporter assay

Luciferase-based assays can be affected by interference of the luciferase reporter system. Therefore, an interference assay was established to identify compounds that might interfere with the nanoluciferase reporter system or luminescence readout. A three-fold serial dilution, starting from 100μM for each compound was prepared onto 1,536-well plates. 5μl of fresh bacterial broth which were incubated at 37 °C overnight was added into each well to give final bacterial concentration of 10^6^CFU/ml. Subsequently, 5μl of nanoluciferase substrate solution (Promega) was added to each well. The plates which were then placed in the dark for 30 min before being for read for luminescence in the Envision Reader (PerkinElmer). The pIC50 ratio between the Caco-2 cell assay and interference assay had to be >1. Compounds with no extracellular-nanoluciferase activity (pIC50 < 4) were considered to be noninterference compounds.

### Computational and statistical analysis

The raw data of the plate reader was imported and analysed in ActivityBase and Spotfire version 10.3.1.18 (TIBCO). The hit cutoff values (Mean ±3 SD), Z’ factor and signal-to-base and percentage of inhibition were calculated automatically by ActivityBase, or manually calculated with equations in Supplementary Table 5. For IC50 measurements, values were normalized and fitted with GraphPad Prism using the following equation: Y = 100 / (1 + [(X / IC50) ^ Hillslope)]; Alternatively, pIC50 values can be calculated as the negative log of the IC_50_ value (pIC50 = -log10(IC50)), which were obtained using the Activity Base XE nonlinear regression bundle. Other data were analysed in Excel (Microsoft Office) and plots were generated using Prism version 6.07 (Graphpad) or IC_50_ calculator tool (ATT Bioquest, https://www.aatbio.com/tools/ic50-calculator).

To analyze the percent inhibition of the primary screen, we constructed an IDBS Activity-Base™ (an established industry software platform) HTS template to calculate the percent inhibition, based on the mean maximum (*n* = 16) and mean minimum (*n* = 16) plate controls, plate signal-to-background (S:B) ratios (mean maximum signal/mean minimum signal), and Z’ factors. The data were exported to TIBCO Spotfire® for visualization to help identify process errors and facilitate quality control analysis. All parameters have been standardized and worldwide used.

The role of high-throughput screening (HTS) is to identify as many small molecule compounds as possible as drug discovery starting points in a fast, practical and cost-efficient manner. High-throughput screening usually starts with a primary screen where small molecules from compound library are usually tested at a single concentration, most commonly 10 μM. From a statistical perspective, the capability of single-point measurements to give an accurate determination of the true activity can be affected by the assay data variability. Hits from the primary screen are determined by selecting compounds, which activity exceeds the hit threshold. Conventionally, the activity hit threshold (also known as hit limit or hit cutoff) is defined as three standard deviations from the mean of the general population. This is based on the notion that in a large compound library where the bulk of the compounds are inactive and compound activities are normally distributed, the mean of the general population is (or is close to) zero percent activity. It also implies that a compound that satisfies that hit threshold has activity which deviates from the bulk of inactive compounds, at the probability of more than 99%^31^.

## Supplementary information

Supplementary information_090425

## Data Availability

All data are presented in the manuscript and raw data are available upon request from the corresponding author. The identified compounds (and associated data) are propriety and owned by GSK, and accessible via the Open Lab Foundation/GSK.
