# Supplementary material for: A three-dimensional high throughput assay identifies novel antibacterial molecules with activity against intracellular Shigella
Source: NPJ Antimicrob Resist. 2025 May 15;3:40. doi: 10.1038/s44259-025-00110-6 (PMC12081684; doi:10.1038/s44259-025-00110-6)
Supplement: Supplementary file 1 — Supplementary information_090425 [file 44259_2025_110_MOESM1_ESM.pdf]

Supplementary figure 1. Comparison of ALP and Sucrose activity at various times after confluence between monolayer and 3-D caco-2 cell models

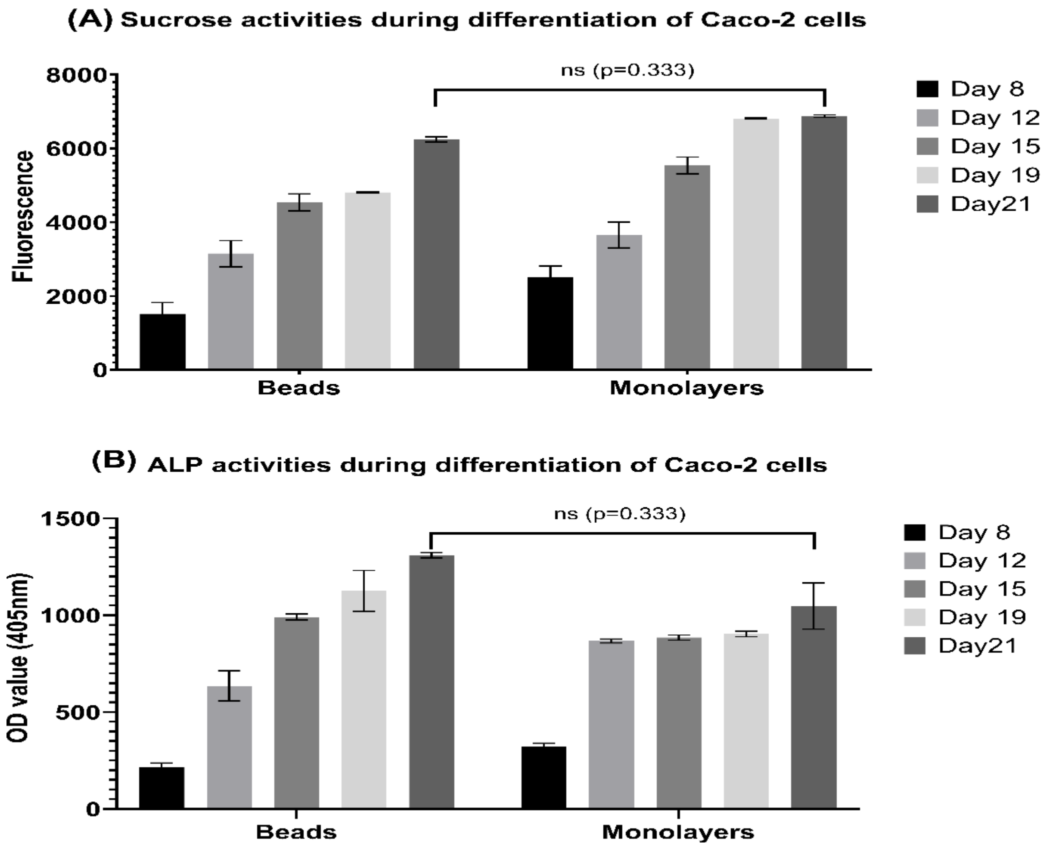

ns: non-significant

Supplementary figure 2. The formation of tight-junctions in Caco-2 cells.

(A) Monolayer of Caco-2 cells; (B) Caco-2 cells on cytodex[3] bead

A

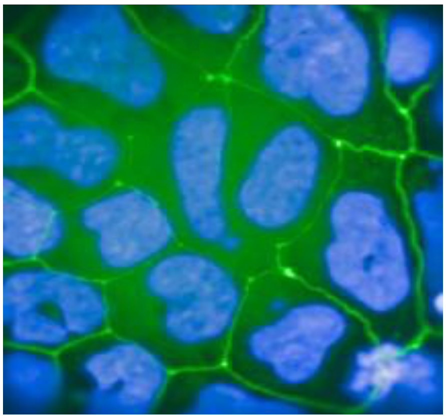

B

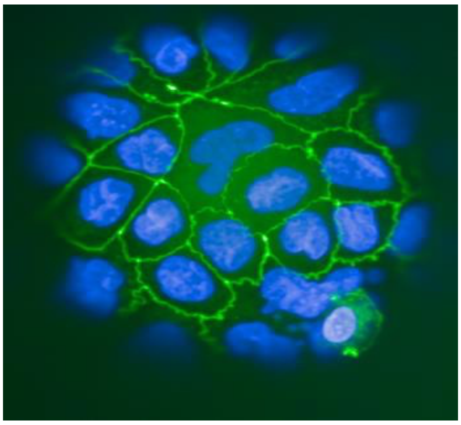

Supplementary figure 3: Primary screening result

Using a z-score cutoff of 0.4, 45 plates were defined as fail and were re-run. The distribution of the z-score for the 1,521 plates is shown here.

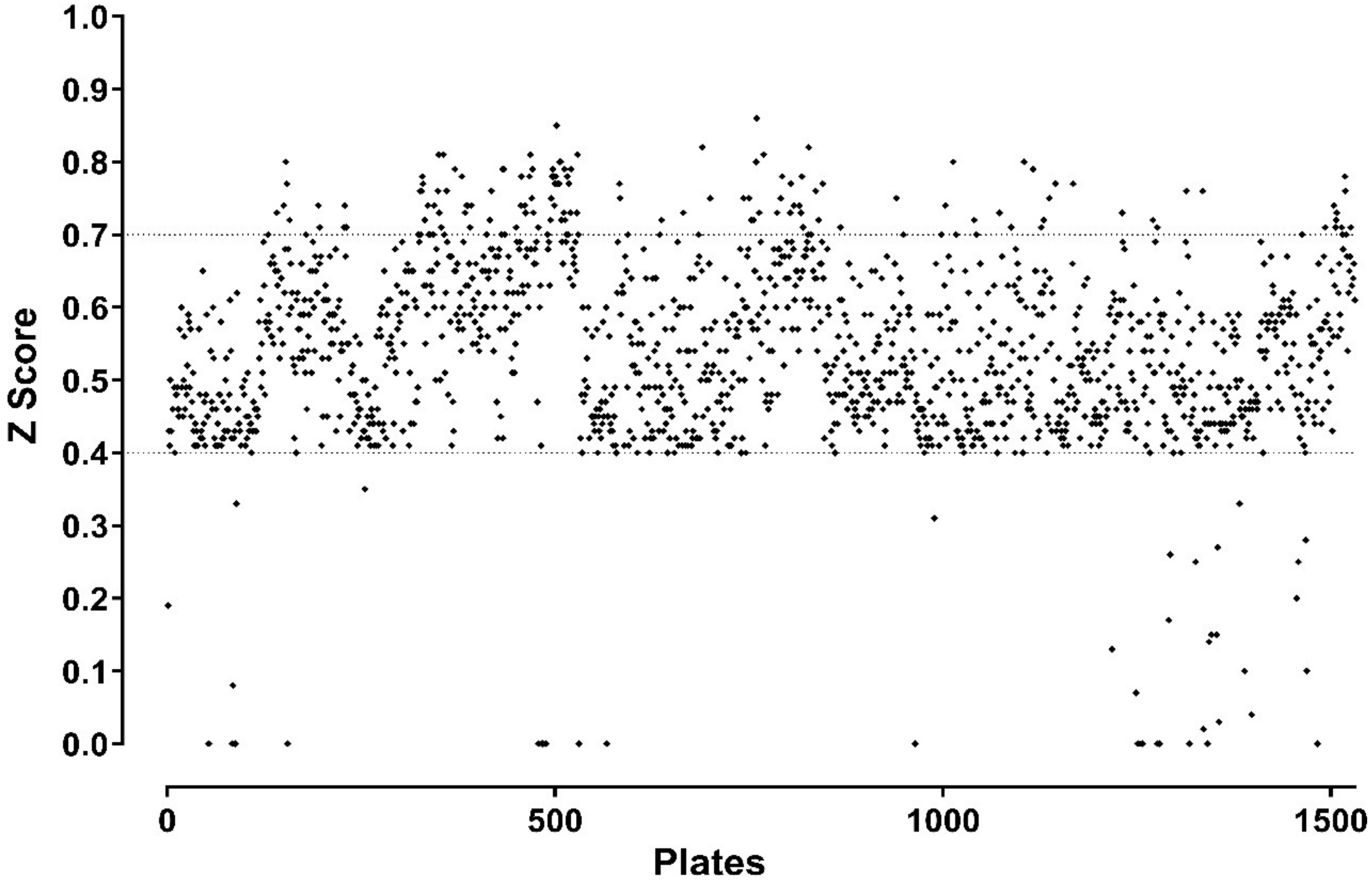

Supplementary table 1. Optimal experimental conditions

	Invasive time (Hours)										Bead number (Beads/ml)		
	4H		6H		8H		10H		Over 16H		1000	2000	4000
MOI	%Cover	Z′	% Cover	Z′	% Cover	Z′	% Cover	Z′	% Cover	Z′	Z'	Z'	Z'
	18.75	< 0	28.13	< 0	59.38	< 0	62.50	< 0	46.88	< 0	ND	ND	ND
5	15.63	< 0	34.38	< 0	50.00	< 0	84.38	< 0	76.19	< 0	ND	ND	ND
	12.50	< 0	28.13	< 0	62.50	< 0	90.63	< 0	56.25	< 0	ND	ND	ND
	15.63	< 0	46.88	< 0	81.25	< 0	59.38	< 0	37.50	< 0	ND	ND	ND
10	12.50	< 0	34.38	< 0	90.63	< 0	81.25	< 0	40.63	< 0	ND	ND	ND
	21.88	< 0	40.63	< 0	87.50	< 0	84.38	< 0	0	< 0	ND	ND	ND
	96.88	0.15	84.38	< 0	96.88	0.06	ND	ND	ND	ND	ND	ND	ND
100	93.75	0.16	100	0.51	100	0.40	ND	ND	ND	ND	ND	ND	ND
	87.50	< 0	96.88	0.35	100	0.46	ND	ND	ND	ND	ND	ND	ND
	100	0.62	100	0.52	96.88	0.31	ND	ND	ND	ND	< 0	0.22	0.61
150	96.88	0.31	100	0.58	93.75	0.23	ND	ND	ND	ND	0.06	0.37	0.61
	100	0.59	100	0.61	100	0.37	ND	ND	ND	ND	< 0	0.34	0.50

*ND: Not done

Supplementary table 2. *Shigella* invasive efficiency

Invasive time	2h	4h	6h
Invasive rate (%)	0.003	0.032	0.083
Shigella per cell (CFU/cell)	0.010	0.044	0.069
Shigella per bead (CFU/bead)	1.748	7.750	15.077

Supplementary table 3. Bacterial strains, cell lines and plasmids

Strain/Cell Line/Plasmid	Phenotype	Source
**Bacterial strains**		
Shigella flexneri 2a 2457T *Castellani and Chalmers* (ATCC® 700930™)	Wild type	American Type Culture Collection
Shigella flexneri SF_nanoluc	S. flexneri ATCC 700930 derivative containing plasmid pMK-RQ_Tac+Nanoluc	
Shigella flexneri 2b 12022 Castellani and Chalmers (ATCC® 12022GFP™)	*S. flexneri* 12022 derivative containing a multicopy vector encoding green florescent protein GFPmut3<br><br>Ampicilin resistance	American Type Culture Collection
**Cell lines**		
Caco-2 cell (ATCC® HTB-37™)	Ethipelial cell	American Type Culture Collection
**Plasmid**		
pMK-RQ_Tac+Nanoluc	pMK-RQ derivative carrying nanoluciferase gene which is exressed under control of Tac promoter<br><br>Kanamycin resistance	Invitrogen

Supplementary table 4. Intra- and inter-assay on IC50 values (μM)

	1st Batch		2nd Batch		IC50 value CV (%)		
**Drugs**	**T1**	**T2**	**T3**	**T4**	**Intra-**	**Inter-**	**Source**
**Meropenem**	0.3486	0.3654	0.4044	0.3993	1.49	6.13	M2574, Sigma-Aldrich
**Tetracycline**	1.6104	1.5576	1.1877	1.1806	0.98	14.51	T7660, Sigma-Aldrich
**Faropenem**	1.2991	1.2299	1.4312	1.444	1.59	6.67	F8182, Sigma-Aldrich
**Ceftriaxone**	0.0151	0.0151	0.0196	0.0196	0.00	12.97	C5793, Sigma-Aldrich
**Azithromycin**	0.569	0.5105	0.6244	0.6242	2.72	8.09	75199, Sigma-Aldrich
**Furazolidone**	1.0673	1.0674	1.3558	1.3678	0.22	12.13	F9505, Sigma-Aldrich
**Moxifloxacin**	0.0252	0.0221	0.0235	0.0235	3.28	4.66	SML1581, Sigma-Aldrich
**Ciprofloxacin**	0.0119	0.0119	0.0156	0.0157	0.16	13.61	17850, Sigma-Aldrich
**Gentamicin**	1.5172	1.2734	1.1708	1.2406	5.82	10.03	G1264, Sigma-Aldrich
**CHIR-090**	0.1895	0.1955	0.2593	0.2593	0.78	14.82	SML3092, Sigma-Aldrich
**PF5081090**	0.0957	0.0919	0.0937	0.0938	1.04	1.43	PZ0194, Sigma-Aldrich

Supplementary table 5: Equations for determining assay performance or sensitivity

Parameters	Equations
Coefficient of Variation	$\%\boldsymbol{CV} = \frac{\sigma}{\mu}\ x\ 100$
Z' factor	$\boldsymbol{Z'} = 1 - \frac{(3\sigma_{max} + 3\sigma_{min})}{\lvert\mu_{max} - \mu_{min}\rvert}$
Signal to background	$\boldsymbol{S}:\boldsymbol{B} = \frac{\mu_{max}}{\mu_{min}}$
Inhibited rate	$\%\boldsymbol{Inhibition} = (1 - \frac{\text{Compound signal} - \text{min signal}}{\text{max signal} - \text{min signal}})\ x\ 100$
pIC50 value	$\mathbf{pIC50} = -\log_{10}(\mathrm{IC50})$

Note: σ, s.d. of the assay signal; μ, mean of the assay signal; max signal, signal with no inhibition; min signal, signal with 100% inhibition.

Supplementary table 6. IC50 comparison between monolayer-based and bead-based assays

**Moxifloxacin IC50 (μM)**		
	Monolayers	Beads
	0.0236	0.0361
	0.0234	0.0327
	0.0228	0.0226
Average	0.0232	0.0305
Mann Whitney test		
P value	0.7	
Exact or approximate P value?	Exact	
P value summary	ns	
Significantly different (P < 0.05)?	No	

Supplementary table 7. Validation set output

Profile		Compound A	Compound B	Compound C	Compound D
**In vitro biology**	Intra (Caco2) IC_{50}(uM)	5.03	6.13	6.85	36.31
	Extra IC_{50} (Res,uM)	8.9	>100	8.7	>100
**Physch-invitro DMPK**	MW	350.3	470.5	410.3	463.4
	$ChromLogD^{7.4}$/ PFI	(1.05^{th}/4.05^{th})<br>2.46 / 5.46	(3.47^{th}/7.47^{th})<br>3.18/ 7.18	(4.6^{th}/7.6^{th})<br>5.69 / 8.69	(4.05^{th}/7.05^{th})<br>4.63 / 7.63
	CAD_Sol (uM)/Fassif	122	5	13	49
	AMP(nm/s)	Low^{th} / nd	Low^{th} / < 3	$High^{th}$ /350	$High^{th}$ /nd
**Safety**	HepG2 IC_{50} (uM)	Not done	>100	100	Not done
	In silico eXP	No alerts	BSEP, MAOA	BSEP, 5HT2C	PLD, HT2Cantg, BSEP, hERG, 5HT2Aantg
	In silico Tox Alerts	Furan toxicity<br>Aromatic amine (hepatotoxicity)	Imidazole tox<br>Aromatic amine (mutagenicity)	Aniline (mutagenicity)<br>Sulphonamide (CYP)	Pyridine tox

Supplementary table 8. Toxicity profiles

Compound	Cluster	Caco2 pIC50	HepG2 pIC50	Selectivity (log) Caco pIC50 vs Cytotox	Structure Alert
1	Series 1	4.63	4.71	0	Aromatic nitro; Aromatic amine; Pyridine tox
2	Series 1	4.69	4.64	0	Aromatic nitro; Thiophene tox
3	Series 2	4.86	nd	nd	Aromatic nitro; Aromatic amine; Secondary sulfonamides
4	Series 2	5.06	<4	>1.06	Aromatic amine; Secondary sulfonamides
5	Singleton 1	4.72	<4	>0.72	No alerts
6	Singleton 2	5.04	<4	>1.04	No alerts
7	Singleton 3	5.49	<4	>1.49	Aromatic amine
8	Singleton 4	4.64	4.26	0.38	Aromatic amine
9	Singleton 5	4.7	4.12	0.58	No alerts
10	Singleton 6	5.07	<4	1.07	Thiazole toxicity
11	Singleton 7	5.24	4.63	0.61	Aromatic nitro
12	Singleton 8	5.80	<4	1.80	No alerts
13	Singleton 9	4.61	<4	>0.61	No alerts
14	Singleton 10	4.61	<4	>0.61	Aromatic amine; Furan toxicity
15	Singleton 11	4.76	<4	>0.76	Aromatic amine; Secondary sulfonamides
